# Enhancing alfalfa yield via micro-nano bubble oxygenation in subsurface drip irrigation: rhizosphere mechanisms unveiled by structural equation modeling

**DOI:** 10.3389/fpls.2025.1702208

**Published:** 2025-11-18

**Authors:** Xuesong Cao, Hexiang Zheng, Yuxiang Wang, Wei Geng, Qianqian Zi, Yaxing Feng

**Affiliations:** 1 Yinshanbeilu Grassland Eco-Hydrology National Observation and Research Station, China Institute of Water Resources and Hydropower Research, Beijing, China; 2 Institute of Water Resources for Pastoral Area, Ministry of Water Resources, Hohhot, China; 3 Comprehensive Support Center of Inner Mongolia Autonomous Region Water Resources Department, Hohhot, China; 4 Water Conservancy Bureau of Ordos City, Ordos, China; 5 Meteorological Bureau of Ordos City, Ordos, China; 6 Agricultural College, Qingdao Hengxing University of Science and Technology, Qingdao, China

**Keywords:** micro-nano bubble oxygenation (MNBO), subsurface drip irrigation (SDI), structural equation modeling (SEM), alfalfa (*Medicago sativa* L.), water-saving irrigation (WSI)

## Abstract

Water scarcity and soil hypoxia are major constraints to forage productivity in arid regions. This study investigated the effects of subsurface drip irrigation with micro-nano bubble oxygenated water (MNBO) on the rhizosphere environment, root physiology, growth, and quality of alfalfa (*Medicago sativa* L. cv. ‘Caoyuan No. 2’) under field conditions in Inner Mongolia. A full factorial experimental design was employed, combining three irrigation levels (20, 25, and 30 mm) and three dissolved oxygen concentrations (1.8, 5.0, and 8.2 mg/L). Structural equation modeling (SEM) was applied to quantify the direct and indirect effects of irrigation and oxygenation on yield formation pathways. Results demonstrated that MNBO irrigation significantly improved soil enzyme activities (catalase by 15–28%; urease by 18–32%), microbial abundance (bacteria, fungi, actinomycetes), and root vitality (27.9–103.6%), thereby promoting plant growth and yield. The optimal treatment (25 mm irrigation with 5.0 mg/L DO) increased dry matter yield by 8.3–30.1%, crude protein by 11.2–11.5%, and crude fat by 18.8–27.6% compared to conventional practices. Structural equation modeling revealed that yield improvement was primarily mediated through enhanced soil biological activity and root functionality, rather than direct irrigation effects. These findings provide a scientifically robust irrigation strategy that synergistically enhances water use efficiency and root zone aeration, offering significant potential to support sustainable forage production in water-limited ecosystems.

## 1 Introduction

Water scarcity poses a major threat to agricultural sustainability in arid and semi-arid regions globally. Inner Mongolia, a key hub for animal husbandry in China, faces limited rainfall, high evaporation rates, and fragile ecosystems (Li and Su, 2017; Gang et al., 2019). These constraints severely limit the production of high-quality forage such as alfalfa (*Medicago sativa* L.), which is vital for the local dairy and livestock sectors. Thus, improving water use efficiency is not only an agronomic priority but also an ecological and economic necessity. Conventional irrigation practices often lead to low water productivity, secondary salinization, and soil compaction (Yan et al., 2020). There is an urgent need to develop and optimize advanced water-saving irrigation technologies that can enhance both the yield and quality of forage crops to strengthen regional agricultural resilience.

Subsurface drip irrigation (SDI) has been widely recognized as an efficient water-saving technique for perennial forage production, as it delivers water directly to the root zone, minimizing evaporation and runoff losses (Zhou et al., 2019; Yao et al., 2021). However, long-term use of buried SDI can lead to challenges such as localized anaerobic conditions and soil compaction around emitters, which inhibit root respiration, microbial activity, and nutrient uptake. These issues are particularly acute in fine-textured soils like those in parts of Inner Mongolia and may constrain the full benefits of SDI (Bhattarai et al., 2008; Naorem et al., 2023). Recent innovations in aeration irrigation, especially the application of micro-nano bubble water (MNBW) in subsurface systems, offer a promising strategy. MNBW exhibits high gas dissolution efficiency, extended stability in water, and strong oxygen-transfer capacity, which can alleviate hypoxia in the root zone and positively influence rhizosphere biogeochemistry (Takahashi, 2005; Liu et al., 2019).

Micro-nano bubble irrigation through subsurface systems has shown potential in enhancing soil microbial communities, enzyme activities, and crop growth in preliminary studies (Bian et al., 2025; Mamun and Islam, 2025). However, most existing research has focused on surface irrigation applications or short-term effects in controlled environments (Zhang et al., 2023). There is still a limited understanding of the soil-plant system’s integrated response to MNBW-SDI—particularly the cascading effects from soil aeration and microbial activation to root vitality and ultimately forage yield and quality under field conditions (Zheng et al., 2025). Furthermore, interactions between irrigation amount and oxygen concentration in subsurface environments are complex and context-dependent, necessitating a systematic approach to identify optimal coupling strategies that are both biologically effective and economically viable for drip irrigation systems (Mattar et al., 2021).

The availability of both water and oxygen in the root zone is a critical determinant of soil health and crop productivity. Soil moisture regulates the diffusion of substrates and oxygen, thereby directly influencing microbial activity and the functioning of soil enzymes such as catalase and urease (Borowik and Wyszkowska, 2016; Lei et al., 2022). Concurrently, oxygen deficiency can shift microbial communities toward anaerobic pathways, suppress the activity of oxidative enzymes, and inhibit root respiration and nutrient uptake (Xing et al., 2025). Recent studies have highlighted that co-optimizing water and oxygen supply can synergistically enhance rhizosphere efficiency. For instance, in subsurface-irrigated systems, supplemental aeration has been shown to significantly increase the abundance of beneficial bacteria and fungi, stimulate enzyme activities, and ultimately improve crop growth and yield (Parameshwarareddy and Dhage, 2022; Ouyang et al., 2023). However, a systematic understanding of how specific water-oxygen combinations quantitatively affect this cascade of processes—from soil microbial and enzymatic responses to root physiology and eventual forage yield—remains limited, particularly under field conditions. This knowledge gap is critical to address for designing precision irrigation strategies in arid lands.

This study employed a multi-factorial orthogonal design combined with structural equation modeling (SEM) to evaluate the effects of micro-nano bubble oxygenation (MNBO) delivered via subsurface drip irrigation on the rhizosphere environment, root physiology, and growth of alfalfa. The objectives were to: (1) evaluate the influence of irrigation amount and oxygen concentration on soil enzyme activities, microbial abundance, and root functionality; (2) identify the optimal water-oxygen combination for enhancing alfalfa growth and quality; and (3) elucidate the direct and indirect mechanisms driving plant and soil responses using SEM. The findings aim to provide a robust scientific basis for designing efficient and sustainable irrigation strategies using MNBO in water-limited regions such as Inner Mongolia.

Catalase activity is a key indicator of soil biochemical process intensity, reflecting the rate of enzymatic decomposition of hydrogen peroxide (Alkorta et al., 2003).

Urease activity is directly involved in the hydrolysis of urea into ammonia, carbon dioxide, and water, serving as an effective indicator of soil nitrogen transformation capacity (Gupta et al., 2023).

Aerobic bacteria, whose growth and reproduction strictly depend on oxygen; an oxygen-deficient environment significantly inhibits their activity and reduces their abundance (Boyd, 2019). A decrease in bacterial scale can slow soil nutrient cycling and reduce the supply of available nutrients.

Fungi are the primary decomposers in acidic soils, participating in the formation and decomposition of humus, ammonification, and nitrification processes, and thrive in well-aerated acidic environments. The abundance of fungi particularly reflects soil fertility and aeration status (Li et al., 2023).

Actinomycetes can degrade various refractory organic compounds, playing important roles in soil fertility, metabolic activity, organic matter transformation, and plant disease control (Javed et al., 2021).

Micro-nano bubble water (MNBW) irrigation ameliorates the root zone oxygen environment, thereby enhancing root morphological and metabolic adaptation and improving water and fertilizer use efficiency (Bhattarai et al., 2005).

## 2 Materials and methods

### 2.1 Experimental site

**Figure 1:**
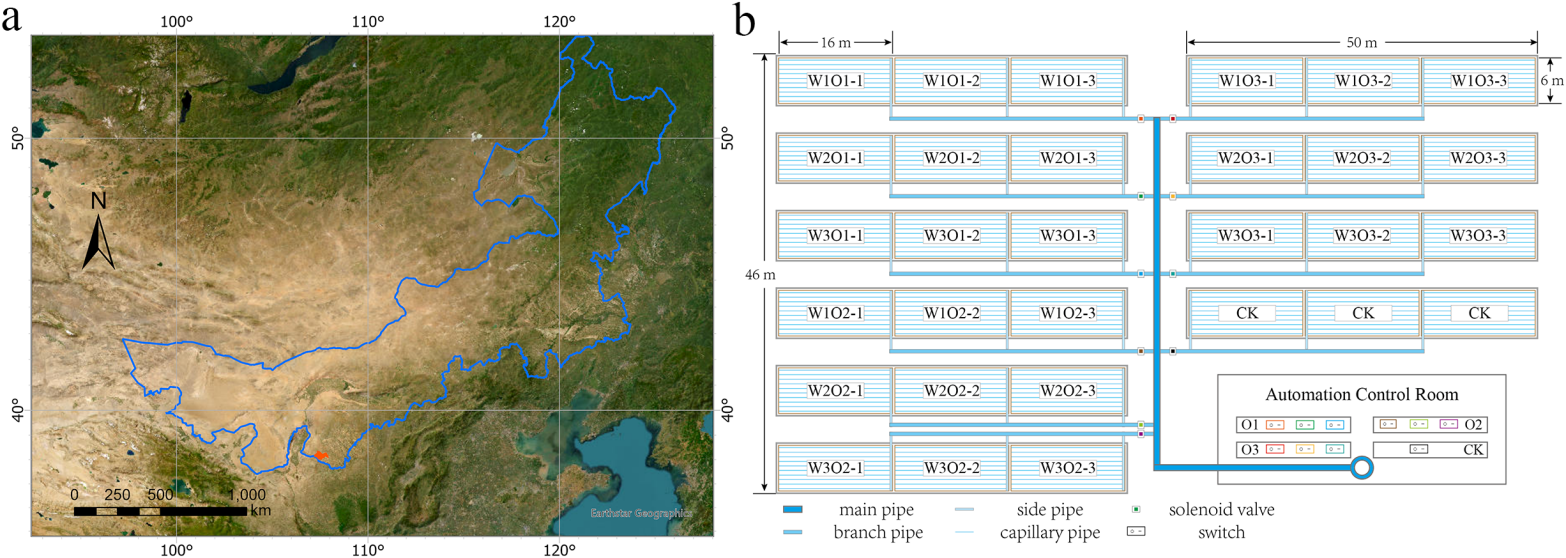
Overview of the experimental area. **(a)** Geographical location of the study region; **(b)** Schematic layout of experimental plots.

The field experiment was conducted at the Water-Saving Experimental Base of Hengfeng Water-Saving Engineering Technology Co., Ltd. in Aoleizhaoqi Town (107° 28’ 8.40”E, 38° 12’ 0.36” N), Otog Front Banner, Ordos City, Inner Mongolia Autonomous Region (**Figure 1A**). This region experiences a warm temperate arid to semi-arid continental climate, with long-term average annual air temperature of 7.9°C, evaporation of 2498 mm, and precipitation of 260.6 mm. Precipitation is unevenly distributed, with over 60% occurring between June and September. The mean frost-free period is 171 days, and the maximum frozen soil depth reaches 1.54 m.

Soil particle-size analysis indicated homogeneous sandy loam texture throughout the 0–100 cm profile. Key physicochemical properties of the topsoil (0–20 cm) were as follows: organic matter of 13.40 g/kg; available phosphorus of 13 mg/kg; available potassium of 176 mg/kg; available nitrogen of 143 mg/kg; bulk density of 1.59 g/cm³; pH of 8.1; saturated water content of 22.04% (w/w); field capacity of 18.58% (w/w); and wilting point of 8.5% (w/w).

### 2.2 Experimental designs

**Figure 2:**
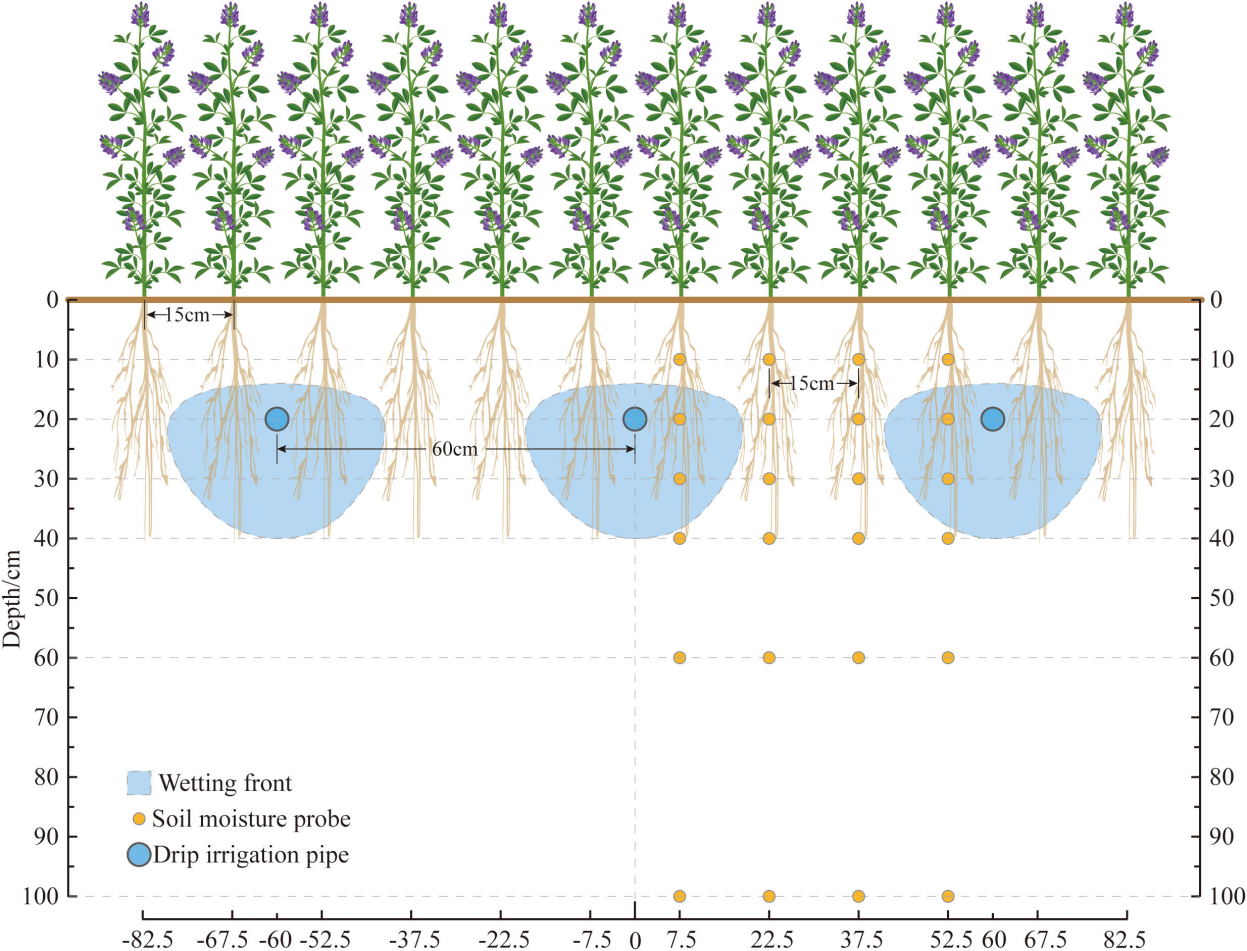
Schematic diagram of the subsurface drip irrigation system with micro-nano bubble (MNB) water for alfalfa cultivation. Field layout: Alfalfa rows (15 cm spacing) serviced by drip tapes buried at 20 cm depth with 60 cm inter-tape spacing. Each tape irrigates four adjacent alfalfa rows. Soil moisture monitoring: Multi-depth sensors installed at 10, 20, 30, 40, 60, and 100 cm depths within the root zone profile.

Alfalfa (*Medicago sativa* L. cv. ‘Caoyuan No. 2’, 4-year-old stand) was hand-sown at a row spacing of 0.15 m. The subsurface drip irrigation system utilized inline emitter drip tape with a wall thickness of 0.4 mm, emitter discharge rate of 2.0 L/h, and emitter spacing of 0.3 m (**Figures 1B**, **2**). Each experimental plot measured 6 m × 50 m, with three replicates, and adjacent plots were separated by a 2-m buffer zone. Each drip tape irrigated four adjacent alfalfa rows, with tapes buried at a depth of 0.2 m and spaced 0.6 m apart (**Figure 2**).

A two-factor, three-level full factorial experimental design was implemented, resulting in nine treatment combinations (3 irrigation levels × 3 dissolved oxygen levels). An additional control treatment (CK) was included (**Table 1**). Irrigation was initiated when root zone soil water content of W2O1 decreased to 60% of field capacity (FC), detail annual irrigation specifications were provided in **Supplementary Table S1**. A control treatment (CK) was included, representing conventional local practices without MNBO, involving four irrigations per year at 80 mm per application. Micro-nano bubbles were generated using a TL-MBG50-A generator (Beijing Zhongnong Tianlu Micro-nano Bubble Water Technology Co., Ltd., China), and DO concentration was measured with an optical probe (YSI Pro20, YSI Inc., USA).

**Table 1:** Full factorial design for MNBO subsurface drip irrigation treatments.

Treatment	Irrigation level^#^ (mm)	DO concentration (mg·L^-^¹)	Annual replicates (2018-2020)
W1O1	20	1.8	3
W1O2	20	5	3
W1O3	20	8.2	3
W2O1	25*	1.8	3
W2O2	25	5	3
W2O3	25	8.2	3
W3O1	30	1.8	3
W3O2	30	5	3
W3O3	30	8.2	3
CK	80	0	3

**^#^: Water amount for each irrigation event.** DO: Dissolved oxygen; *: Irrigation triggered when W2O1 treatment field capacity drops to 60%; CK: Irrigated with 80 mm of normal water 4 times a year, which was based on the conventional irrigation practices of local herders. Annual replicates indicate repeated measurements across the 2018–2020 growing seasons.

Urea was applied at the first irrigation following stem elongation at a rate of 8 kg N ha^-^¹ for the first and second harvests, and 5 kg N ha^-^¹ for the third harvest via fertigation. Urea was dissolved uniformly in the fertilizer tank at the system head and delivered directly to the root zone with irrigation water. The alfalfa was harvested three times per year at the early flowering stage to optimize yield and forage quality. The typical harvesting schedule across the three growing seasons (2018-2020) was as follows: the first harvest in mid-to-late May, the second harvest in early-to-mid July, and the third harvest in late September. The regreening period for the first crop commenced in early April each year.

### 2.3 Observational indicators and methods

#### 2.3.1 Soil moisture monitoring

Within each plot, four vertical arrays of soil moisture sensors were installed in the inter-row spaces between alfalfa rows serviced by two adjacent drip tapes. Sensors were placed at depths of 10, 20, 30, 40, 60, and 100 cm, totaling 24 automated probes per treatment plot (**Figure 2**). Volumetric soil water content was recorded at 5-minute intervals using an automated monitoring system.

#### 2.3.2 Meteorological data

**Figure 3:**
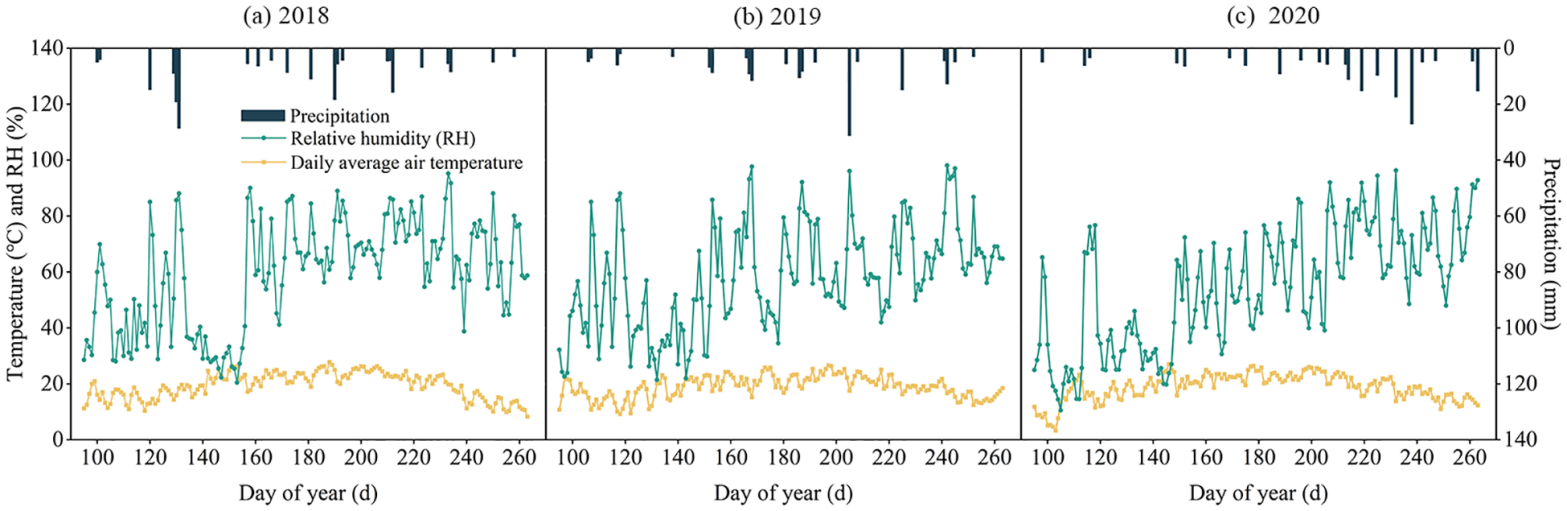
The average daily air temperature, relative humidity and precipitation during the growth period of alfalfa in 2018 **(a)**, 2019 **(b)** and 2020 **(c)**.

Meteorological parameters including daily air temperature, precipitation, wind speed, relative humidity, atmospheric pressure, and wind direction were monitored throughout the experimental period (2018-2020) using an automated weather station (HOBO U30, Onset Computer Corporation, USA) installed at the site. Growing season precipitation measured 207.86 mm (2018), 177.70 mm (2019), and 183.50 mm (2020), representing 79.8%, 68.2%, and 70.5% of respective annual totals. As showed in **Figure 3**.

#### 2.3.3 Soil enzyme activity

Soil samples were collected during the flowering period of the second crop of alfalfa each year to measure soil enzyme activity. Soil samples were collected to obtain a representative composite sample of the primary root zone (0–40 cm depth) for each replicate. Within the area serviced by a single drip tape (four rows of alfalfa), multiple soil cores were taken randomly from the rhizosphere using an auger. This sampling covered a range of horizontal distances from the drip tape to ensure the integration of potential spatial heterogeneity. All cores from the same replicate were thoroughly mixed into one composite sample prior to analysis. This approach was designed to measure the average soil enzyme activity across the entire root zone system. Soil catalase activity was determined using the permanganate titration method according to Alef and Nannipieri (1995). Hydrogen peroxide enzyme activity was determined using the titration method (titration with 0.1 mol/L standard KMnO_4_ solution). Soil hydrogen peroxide enzyme activity was expressed as the milliliters of KMnO_4_ solution consumed per gram of dry soil, with units of ml/g. Urease activity was determined using the phenol-sodium hypochlorite colorimetric method. Soil urease activity was expressed as the mass of NH_3_-N produced per gram of soil after 24 hours (1 day), with units of mg/(g•d).

#### 2.3.4 Soil microbial population

Soil microbial populations were measured during the second flowering period each year. Root-zone soil was collected by excavating the entire root system of alfalfa plants. After shaking off large, loose soil clumps, the soil closely associated with the roots (adhering to roots from approximately 0 to 60 cm depth) was carefully collected using a soft-bristled brush. This soil, representing a composite sample from the entire root profile, was thoroughly homogenized. Three replicate samples were taken for each treatment. The collected soil samples are sealed in sterile plastic bags and stored at 4°C for microbial quantity determination. Soil microbial populations (bacteria, fungi, and actinomycetes) were quantified using the standard serial dilution plate count method (Zibilske, 1994). Briefly, soil sample (0.1 ml of bacterial suspension) inoculated into three consecutive dilutions, each diluted three times. Bacteria were cultured on beef broth agar medium, fungi on Martin’s medium, and actinomycetes on MMGA (Modified Gao’s Medium). All soil samples were randomly collected within the plot, with three replicates per treatment. All operations were performed under sterile conditions to prevent contamination.

#### 2.3.5 Alfalfa root activity

Root samples were collected during the regrowth, stem elongation, branching, and flowering stages of the second alfalfa harvest each year. Root activity of individual plants was determined using the triphenyltetrazolium chloride (TTC) reduction assay (Knievel, 1973). Root activity was expressed as the mass of triphenylformazan (TTF) produced per gram of fresh root per hour (mg g^-^¹ h^-^¹).

#### 2.3.6 Alfalfa plant height and yield

Alfalfa was harvested at flowering, with three cuttings per year. Alfalfa plant height, yield, and quality were measured using the sampling method. The sample area is 1 m × 1 m. Plant height is measured using a caliper, with three measurements taken per sample, and the average value is recorded. After cutting, the fresh weight of alfalfa is weighed. The fresh grass samples are then placed in an oven, subjected to high-temperature killing at 105°C for 30 minutes, followed by temperature adjustment to 65°C, and dried under constant temperature conditions for 48 hours. After cooling, the dry weight is measured.

### 2.4 Statistical analysis and visualization

Statistical analyses were performed using SPSS 25.0 (IBM Corp., USA). One-way analysis of variance (ANOVA) was used to determine the significance of differences among treatments. Multiple comparisons between groups were conducted using Duncan’s test when the ANOVA indicated significant differences. Pearson correlation analysis was employed to evaluate bivariate relationships between key variables. Structural Equation Modeling (SEM) was developed and analyzed using AMOS (IBM SPSS Amos) to explore complex causal pathways among observed variables. All graphs were generated using Origin 2024 (OriginLab Corp., USA) and further refined for clarity and presentation using Adobe Illustrator 2024 (Adobe Inc., USA).

## 3 Results

### 3.1 Effects of micro-nano bubble water subsurface drip irrigation on soil enzyme activities

#### 3.1.1 Catalase activity

**Figure 4:**
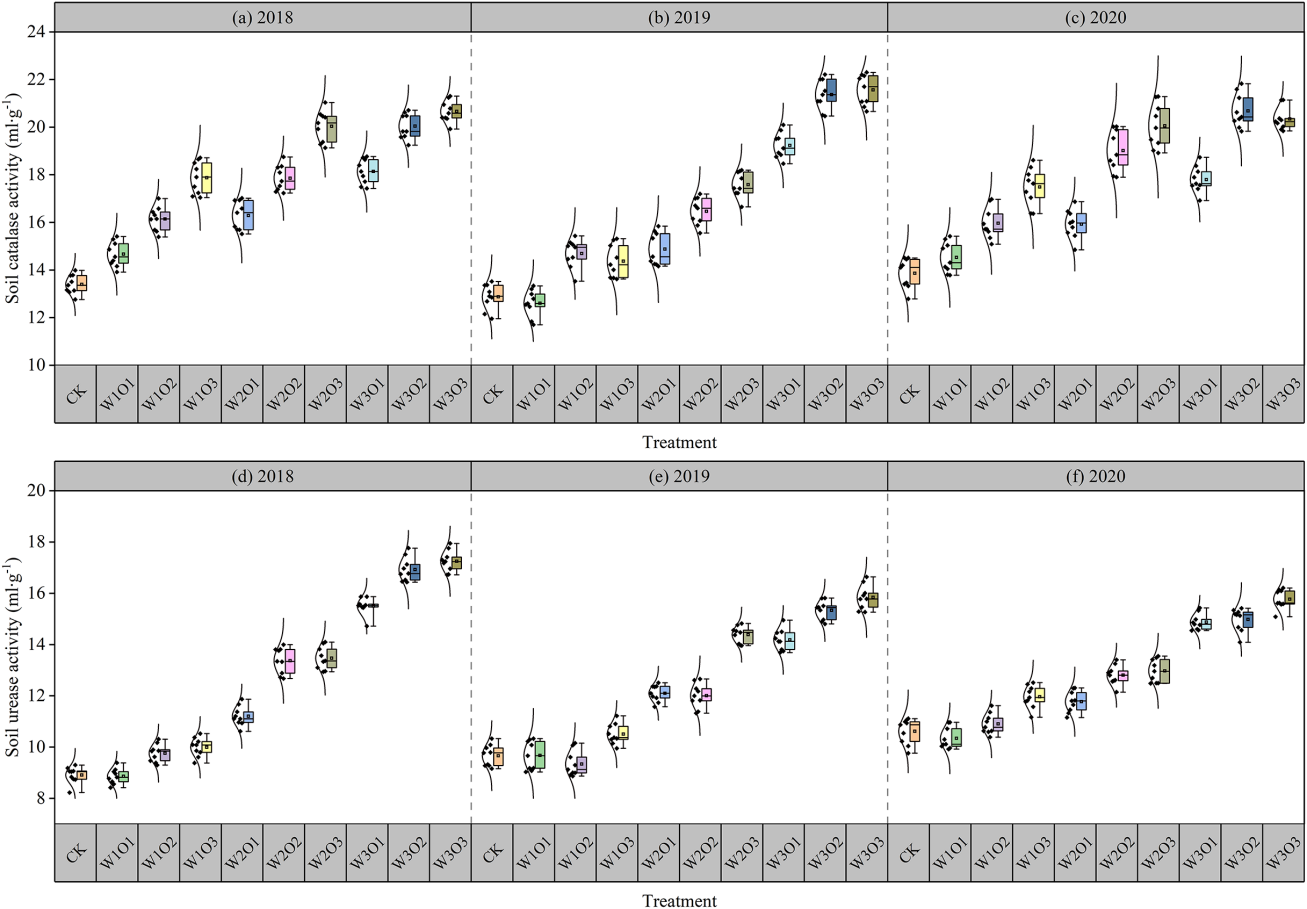
The enzyme activity of the second-cut flowering stage. Catalase in 2018 **(a)**, 2019 **(b)** and 2020 **(c)**. Urease in 2018 **(d)**, 2019 **(e)** and 2020 **(f)**.

From 2018 to 2020, micro-nano bubble water subsurface drip irrigation significantly enhanced catalase activity in the rhizosphere soil of alfalfa (**Figures 4A-C**; the second-cut flowering stage of alfalfa). Under the same irrigation quota, enzyme activity increased significantly with higher dissolved oxygen levels, indicating that oxygen-enriched irrigation can enhance microbial metabolic activity by improving root zone aeration. Under fixed dissolved oxygen conditions, enzyme activity also increased with higher irrigation quota, aligning with the moisture-mediated promotion of enzyme activity. The catalase activity in the control group (CK) was significantly lower than that in all micro-nano bubble treatments, indicating that conventional irrigation regime limits soil biochemical processes. During the study period, the W3O3 treatment (high irrigation and high dissolved oxygen) yielded the highest enzyme activity which was 39.5 ~ 42.4% higher than CK.

#### 3.1.2 Urease activity

As shown in **Figures 4D-F**, micro-nano bubble treatments significantly increased urease activity in the alfalfa rhizosphere from 2018 to 2020 (the second-cut flowering stage of alfalfa). Under the same irrigation conditions, urease activity increased with higher dissolved oxygen levels, supporting the view of that oxygen availability promotes the activity of nitrogen-cycling microorganisms. Under fixed dissolved oxygen conditions, increased irrigation also enhanced enzyme activity, consistent with the moisture–microbe interaction mechanism proposed. The urease activity in the CK group was consistently the lowest and significantly lower than that in micro-nano bubble treatments, reflecting the constraints of conventional irrigation regime on nitrogen transformation efficiency. During the study period, the W3O3 treatment (high irrigation and high dissolved oxygen) yielded the highest enzyme activity which was 40.1~ 54.2% higher than CK.

### 3.2 Effects of micro-nano bubble water subsurface drip irrigation on soil microorganisms

#### 3.2.1 Effects on soil bacteria

**Figure 5:**
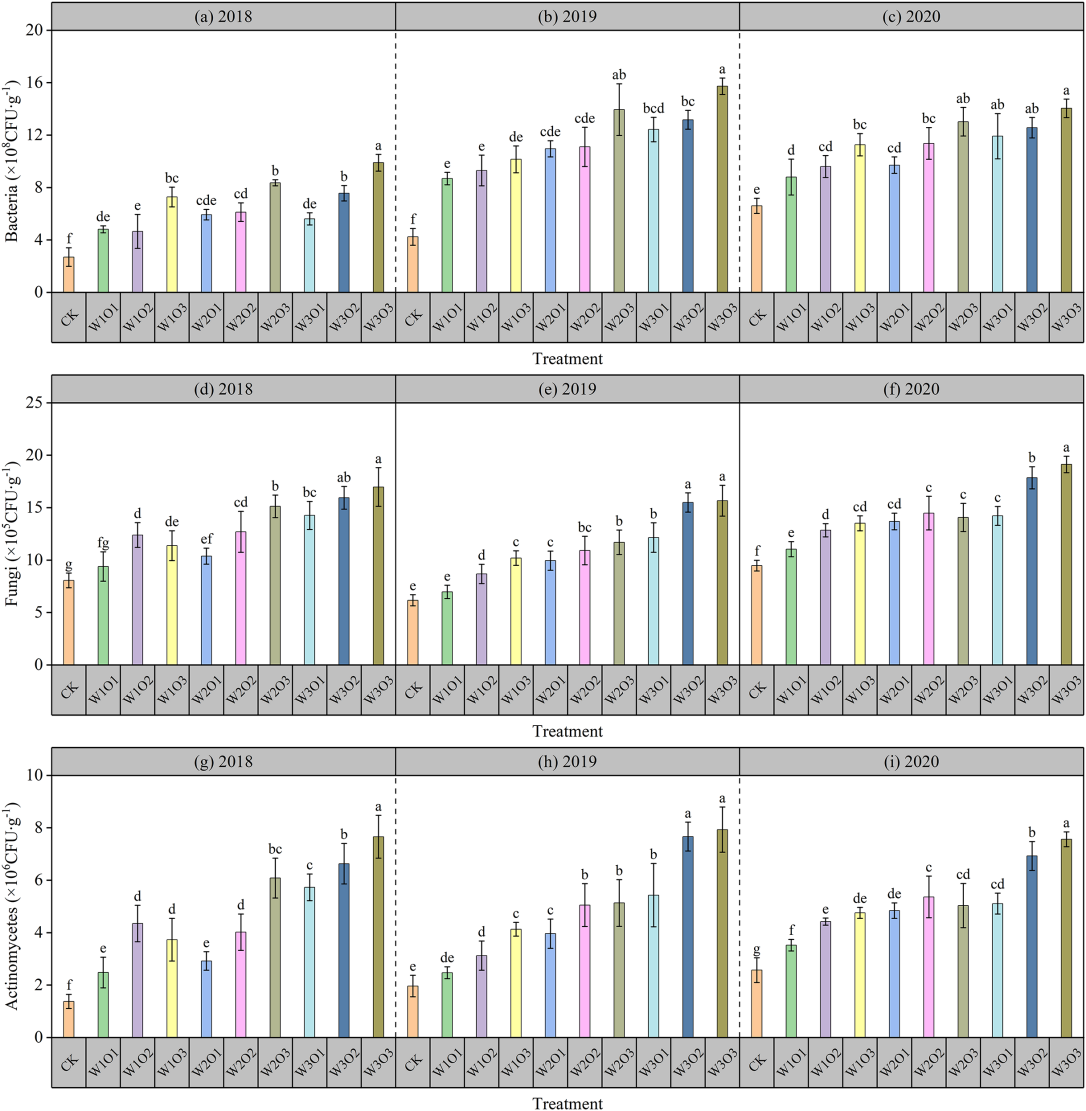
Soil microorganisms abundance during the flowering stage of the second-cut alfalfa. Bacteria in 2018 **(a)**, 2019 **(b)** and 2020 **(c)**. Fungi in 2018 **(d)**, 2019 **(e)** and 2020 **(f)**. Actinomycete in 2018 **(g)**, 2019 **(h)** and 2020 **(i)**.

As shown in **Figures 5A-C**, micro-nano bubble water subsurface drip irrigation significantly increased soil bacterial counts compared to CK. Under a fixed irrigation quota, bacterial numbers increased with higher dissolved oxygen levels, indicating that elevated oxygen promotes the reproduction of aerobic bacteria. Under constant dissolved oxygen conditions, bacterial counts also rose with increasing irrigation quota. Although high irrigation may reduce soil aeration to some extent, the higher soil moisture and improved root-wetting zone matching created a more suitable humid environment, thereby favoring bacterial growth.

Specifically, the results from 2018 to 2020 showed a consistent trend, while the highest bacterial count in the micro-nano bubble water treatment (W3O3) increased by 112.68% to 271.15% compared to CK. Although high irrigation may reduce soil aeration to some extent, the higher soil moisture content and improved matching degree of root zone wetting area create a more suitable humid environment, thereby promoting bacterial growth.

#### 3.2.2 Effects on soil fungi

As shown in **Figures 5D-F**, micro-nano bubble water subsurface drip irrigation significantly increased soil fungal counts compared to CK. Under a fixed irrigation quota, fungal numbers initially increased and then decreased with rising dissolved oxygen levels: moderate oxygen improvement enhanced soil aeration and promoted fungal reproduction, but excessively high oxygen increased gas flow disturbance to hyphae, inhibiting fungal numbers and metabolic enzyme activity. Under constant dissolved oxygen conditions, fungal counts slightly increased with higher irrigation quota, as increased soil moisture optimized root-water matching and created a more suitable humid environment, partially offsetting the negative effects of reduced aeration.

Specifically, the results from 2018 to 2020 showed a consistent trend, while the highest fungi count in the micro-nano bubble water treatment (W3O3) was found increasing by 101.83% to 154.04% compared to CK. These results indicate that micro-nano bubble water subsurface drip irrigation can significantly promote fungal abundance by regulating the soil aeration environment, thereby enhancing soil organic matter decomposition and nutrient use efficiency.

#### 3.2.3 Effects on soil actinomycetes

As shown in **Figures 5G-I**, the effect of micro-nano bubble water subsurface drip irrigation on soil actinomycete counts was similar to that on fungi. Compared to CK, the micro-nano bubble water treatment significantly increased actinomycete abundance. Under a fixed irrigation quota, actinomycete numbers initially increased and then decreased with rising dissolved oxygen levels: moderate oxygen improvement enhanced soil aeration and promoted actinomycete growth, but excessively high oxygen increased gas flow disturbance to the Actinomycetes, inhibiting their numbers and metabolic activity. Under constant dissolved oxygen conditions, actinomycete counts slightly increased with higher irrigation quota, as increased soil moisture optimized root-water matching and humid-air balance, partially mitigating the negative effects of reduced aeration.

Specifically, the results from 2018 to 2020 showed a consistent trend, while the highest actinomycete count in the micro-nano bubble water treatment (W3O3) increased by 191.41% to 456.99% compared to CK. The results demonstrate that micro-nano bubble water subsurface drip irrigation can significantly increase actinomycete abundance by improving the soil aeration environment, thereby promoting organic matter decomposition, nutrient transformation, and soil fertility enhancement.

### 3.3 Effects of micro- and nano-bubble water subsurface drip irrigation on root vitality

**Figure 6:**
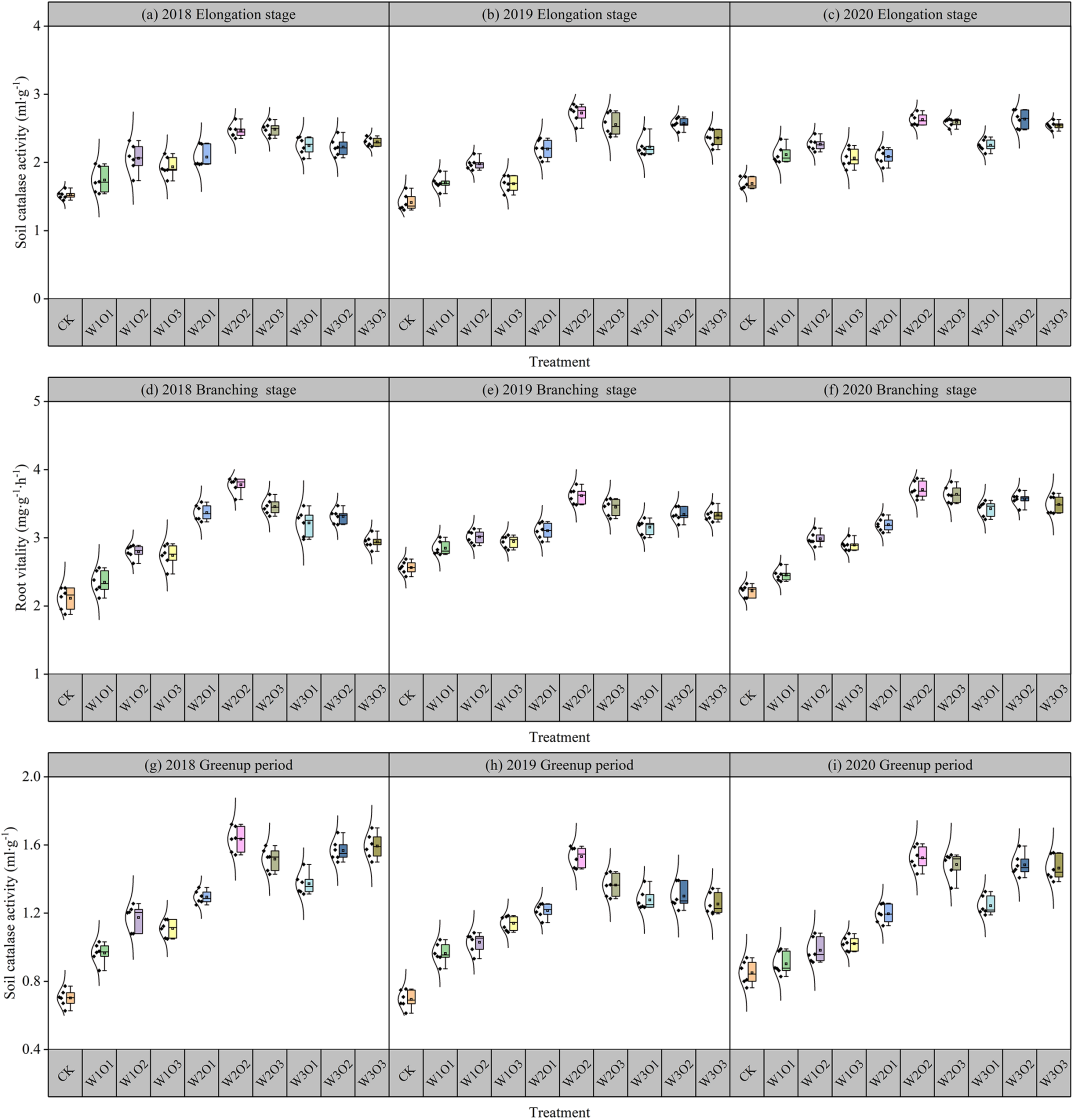
Root vitality of alfalfa during different growth stages in the second-cut harvest. Elongation stage of 2018 **(a)**, 2019 **(b)** and 2020 **(c)**. Branching stage of 2018 **(d)**, 2019 **(e)** and 2020 **(f)**. Greenup period of 2018 **(g)**, 2019 **(h)** and 2020 **(i)**.

Data from 2018 to 2020 showed that the root activity of all treatment groups followed a basic trend: branching stage > jointing stage > flowering stage (**Figure 6**). The root activity under MNBW irrigation treatment was always higher than that of the non-aerated control group, among which the high aeration treatment (W2O2) had the most significant improvement effect. During the jointing stage, it increased by 146.18% to 172.43% compared to the control group. During the branching stage, it increased by 261.63% to 277.65% compared to the control group. During the flowering stage, it increased by 52.46% to 63.44% compared to the control group.

At a fixed dissolved oxygen (DO) concentration, root activity exhibited a unimodal response to increasing irrigation volume, initially rising and subsequently declining. This pattern suggests that moderate irrigation enhances root–water coupling, whereas excessive moisture negatively affects metabolic function. Similarly, under a constant irrigation amount, root vitality showed an initial increase followed by a slight decrease as DO levels rose, indicating that optimal micro-nano bubble (MNB) aeration mitigates oxygen limitation, while super-saturated DO may induce physiological inhibition due to excessive gaseous perturbation. The improvement in root activity under MNB-enriched irrigation is mainly due to enhanced oxygen supply, which stimulates the release of oxidative root exudates that help detoxify reduced compounds in the rhizosphere. In contrast, anaerobic conditions suppress oxygen-dependent root functions, promote denitrification and reduction of iron and sulfur, and may eventually lead to root rot and functional impairment.

### 3.4 Effect of subsurface drip irrigation with micro-nano bubble water on biomass

#### 3.4.1 Response of plant height to water-air coupling

**Figure 7:**
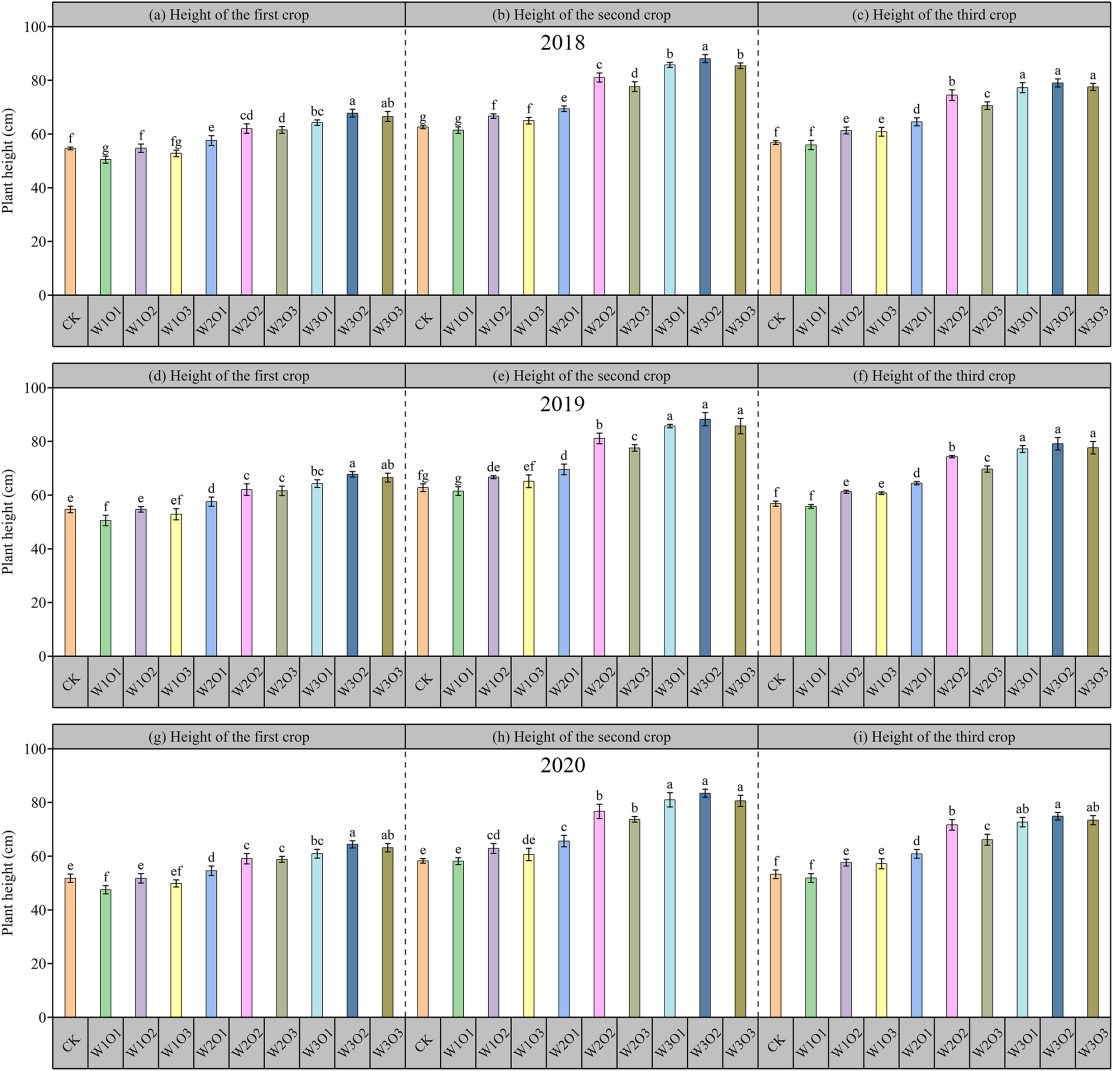
Plant height of alfalfa across three harvests from 2018 to 2020. First, second, and third harvest of 2018 **(a-c)**; 2019 **(d-f)**; and 2020 **(g-i)**.

As illustrated in **Figure 7**, plant height of alfalfa during the flowering period from 2018 to 2020. Overall, the nine datasets exhibited generally consistent trends. No significant difference (P > 0.05) was observed between the control and the W1O1 treatment, though plant height in both was moderately lower than that in the W1O2 and W1O3 treatments. As the irrigation volume increased to medium (W2) and high (W3) levels, plant height showed a significant increase with increasing irrigation amount (P <0.05). Under the same irrigation level, plant height initially increased significantly with rising dissolved oxygen (DO) concentration. However, the plant height under the O3 treatment was either slightly lower than or not significantly different from that under O2, suggesting the existence of a DO threshold beyond which oxygen no longer acts as a limiting factor for plant height, and excessive DO may even inhibit root nutrient uptake efficiency. Therefore, it can be preliminarily concluded that irrigation volume has a greater influence on plant height than dissolved oxygen concentration, with irrigation amount being the dominant factor governing plant height growth.

#### 3.4.2 Response of dry matter accumulation to water-air coupling

**Figure 8:**
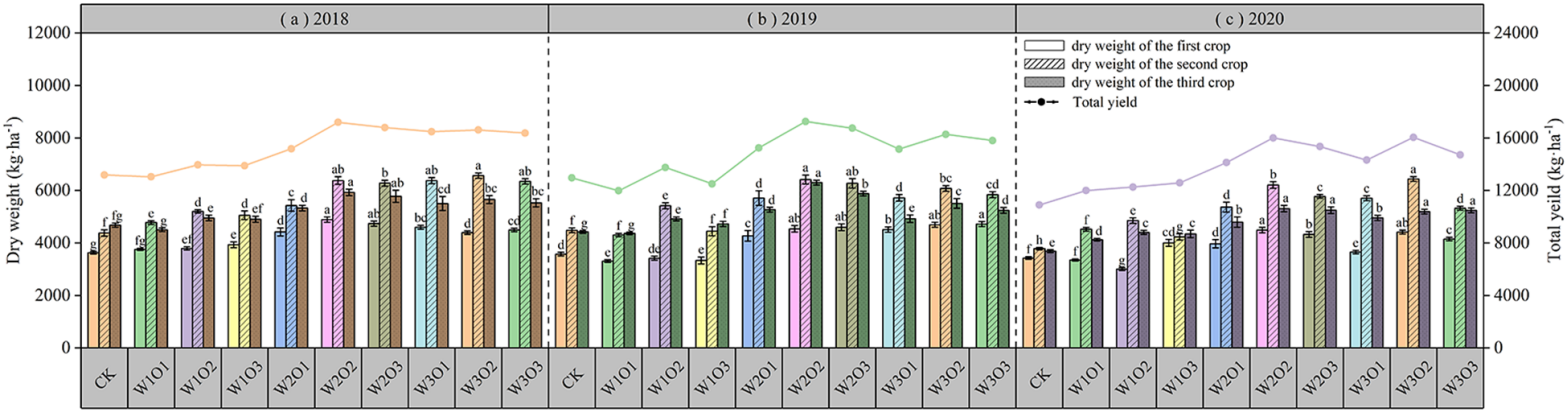
Yield of alfalfa across three harvests from 2018 to 2020. 2018 **(a)**; 2019 **(b)**; and 2020 **(c)**.

As shown in **Figure 8**, micro-nano bubble irrigation significantly improved the dry matter yield of alfalfa: under a fixed irrigation volume, appropriate increase in dissolved oxygen (DO) concentration led to higher yield, but further oxygen increase resulted in diminishing returns; under constant DO conditions, raising the irrigation amount also enhanced yield, yet continuing increase similarly showed a trend of diminishing marginal gains. In 2018 and 2019, the W2O2 treatment resulted in the highest dry matter yield, while in 2020, the W3O2 treatment produced the highest yield, though the W2O2 treatment was slightly lower and not significantly different (P > 0.01). Compared with the control, the W2O2 treatment increased dry matter yield by 23.34% to 31.95%. These results indicate that optimizing the water–air combination can improve the root-zone moisture environment, enhance microbial and root activity, and promote nutrient transformation and uptake.

### 3.5 Correlation and structural equation modeling analysis

#### 3.5.1 Correlation analysis

**Figure 9:**
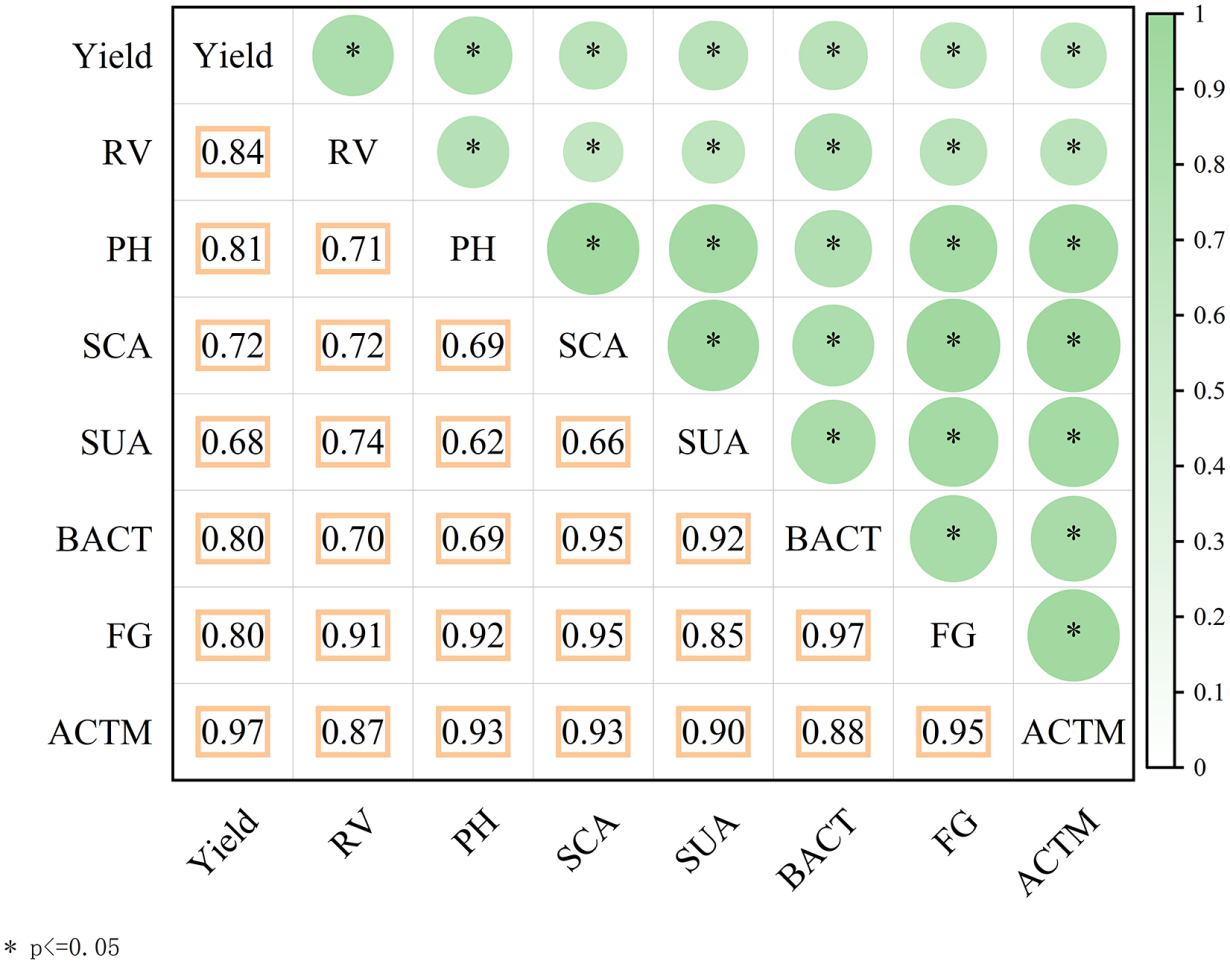
Correlation matrix illustrates relationships among soil properties, plant growth, and yield indicators under micro-nano bubble oxygenated subsurface drip irrigation. Significant pairwise correlations (p ≤ 0.05) are shown. Yield demonstrated strong positive correlations with root vitality (RV), plant height (PH), microbial communities (BACT, FG, ACTM), soil catalase activity (SCA), and soil urease activity (SUA).

Correlation analysis was employed to quantify bivariate relationships among key indicators of soil properties, plant growth, and yield under micro-nano bubble subsurface drip irrigation. The correlation matrix (significant at p ≤ 0.05) is presented in **Figure 9**.

Yield showed significant positive correlations with all measured variables. Specifically, strong positive relationships were identified between yield and root vitality (RV), plant height (PH, r = 0.81), as well as microbial communities (bacteria—BACT, fungi—FG, and actinomycetes—ACTM), with correlation coefficients ranging from R² = 0.81 to 0.97. Yield was also highly correlated with soil catalase activity (SCA) and soil urease activity (SUA). These strong correlations suggest that micro-nano bubble subsurface drip irrigation enhances soil biological activity and plant physiological performance, collectively contributing to improved yield.

#### 3.5.2 Structural equation modeling analysis

In this study, a Structural Equation Modeling (SEM) was developed using AMOS to analyze relationships among key variables—irrigation, oxygen concentration, soil enzymes (catalase, urease), soil microbes (bacteria, fungi, actinomycetes), plant traits (height, root vitality), and yield. The model demonstrated acceptable to good fit to the data (CMIN/df = 4.32, GFI = 0.853, RMSEA = 0.041), supporting the proposed conceptual framework.

**Figure 10:**
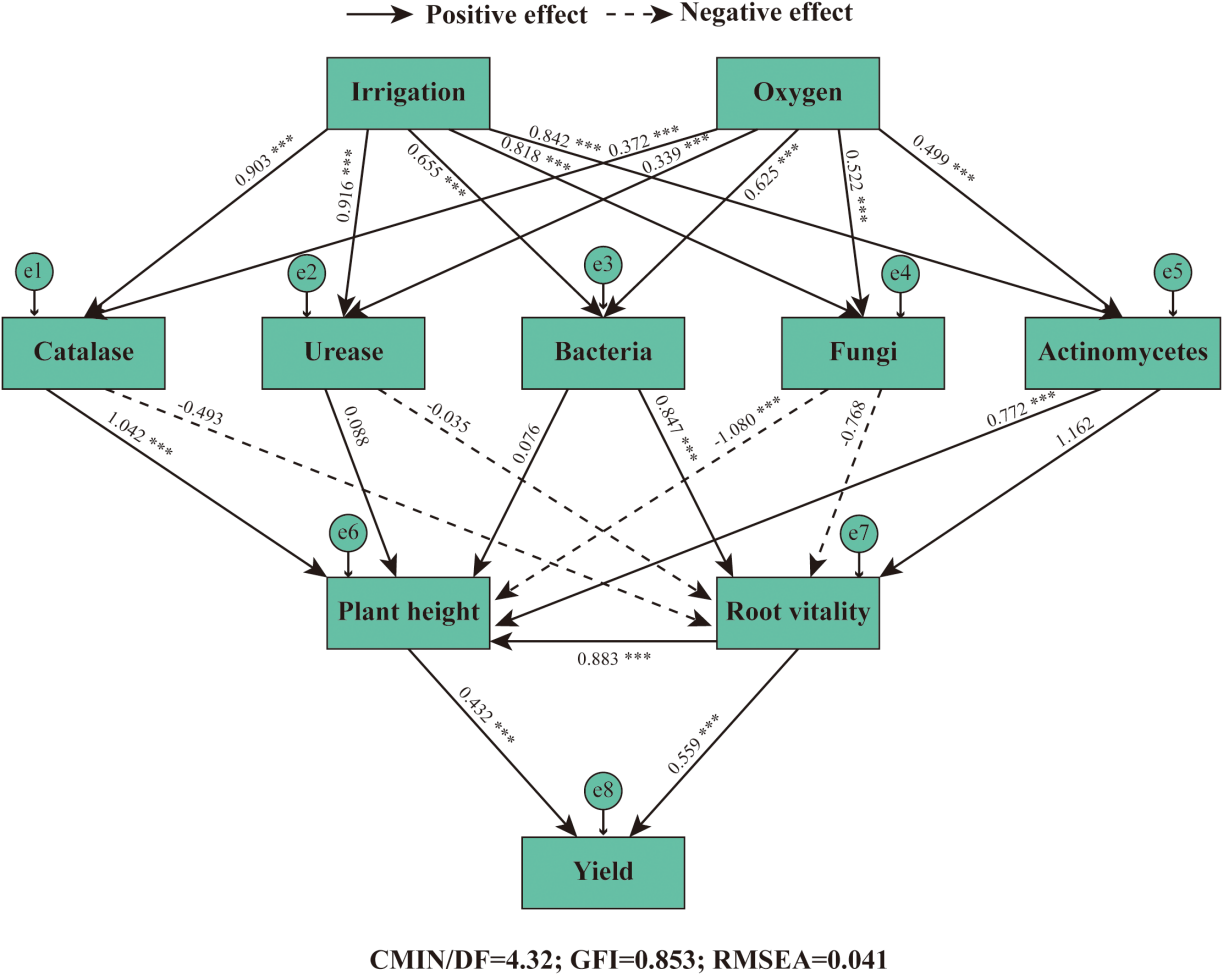
Structural equation modeling (SEM) depicting the pathways through which irrigation and oxygen influence alfalfa yield via rhizosphere biological mechanisms. Solid arrows represent significant positive pathways (P <0.05), while dashed arrows indicate significant negative pathways (P <0.05). Values adjacent to arrows are standardized path coefficients. *** indicates p < 0.001. Irrigation: Subsurface drip irrigation treatments; Oxygen: Micro-nano bubble oxygen concentration; Soil Enzymes: Catalase activity, Urease activity; Soil Microbes: Bacterial abundance, Fungal abundance, Actinomycetes abundance; Plant Traits: Plant height, Root vitality; Yield: Alfalfa dry matter yield. Model fit indices: CMIN/df = 4.32, GFI = 0.853, RMSEA = 0.041, indicating acceptable model fit.

The path analysis revealed a complex web of interactions (**Figure 10**). Irrigation exerted strong direct positive effects on rhizosphere biology, with highly significant standardized path coefficients of 0.903*** for catalase, 0.916*** for urease, 0.655*** for bacteria, 0.818*** for fungi, and 0.832*** for actinomycetes. Micro-nano bubble oxygen concentration displayed an additional, independent positive influence on all these biological parameters (path coefficients ranging from 0.339*** to 0.625***), underscoring its critical role beyond water application alone.

Notably, the model uncovered distinct mediation pathways through which these improved soil conditions translated into yield gains. Soil microbial communities influenced plant growth in contrasting ways. Fungal abundance exhibited a significant negative effect on plant height (-1.080), whereas actinomycetes had a strong positive effect (0.772). This suggests a potential competitive inhibition or pathogen pressure from certain fungi, counteracted by the beneficial contributions of actinomycetes. Furthermore, root vitality was primarily enhanced by bacterial abundance (0.847), highlighting the importance of bacteria in promoting root physiological function. Ultimately, yield was positively and almost equally driven by both increased plant height (0.432) and enhanced root vitality (0.559***).

These results demonstrate that the beneficial effect of micro-nano bubble irrigation on yield is predominantly indirect, mediated through the creation of a favorable rhizosphere environment. The treatment first stimulates microbial and enzymatic activity, which then modulates plant growth and physiology through specific, and sometimes opposing, pathways. The strong negative path from fungi to plant height is a particularly insightful finding, suggesting that future management strategies could aim to suppress specific fungal groups while promoting beneficial bacteria and actinomycetes. This SEM analysis confirms that yield enhancement is not a simple direct effect but the culmination of a cascade of improvements in soil biological health, which in turn boost root function and canopy development, leading to superior crop performance.

## 4 Discussion

The development of water-saving irrigation technologies is essential for sustainable forage production in arid and semi-arid regions such as Inner Mongolia. This study demonstrated that subsurface drip irrigation with micro-nano bubble water (MNBW) significantly enhances alfalfa yield by 23.34–31.95% compared to the conventional irrigation control (CK), with the optimal combination identified as 25 mm irrigation volume and 5.0 mg/L dissolved oxygen (DO). align with findings that oxygenated irrigation mitigates soil hypoxia, particularly in subsurface drip systems (Bhattarai et al., 2005; Mattar et al., 2021).

Improvements in soil health were central to this response. MNBW irrigation significantly increased in catalase (39.5–42.4%) and urease (40.1–54.2%) activities compared to CK. As established biomarkers of soil health, the enhancement of these enzymes—catalase alleviating oxidative stress and urease facilitating nitrogen mineralization—indicates a robust biochemical environment (Alkorta et al., 2003; Gupta et al., 2023). Strong positive correlations (r = 0.62-0.97) among microbial abundance, enzyme activities, and root vitality suggest that MNBW irrigation fosters a synergistic rhizosphere environment where enhanced microbial activity supports nutrient cycling and plant growth (Liu et al., 2019; Javed et al., 2021), which is particularly vital in biologically constrained arid soils (Naorem et al., 2023).

To elucidate the causal pathways, Structural Equation Modeling (SEM) was employed. The model demonstrated acceptable fit (CMIN/df = 4.32, GFI = 0.853, RMSEA = 0.041) and revealed that irrigation and oxygen exerted strong direct positive effects on rhizosphere biology (path coefficients: 0.903*** for catalase; 0.916*** for urease). Crucially, the model uncovered distinct microbial mediation pathways: fungal abundance negatively impacted plant height (-1.080), whereas actinomycetes showed a positive effect (0.772). Root vitality was primarily enhanced by bacteria (0.847), and yield was ultimately driven by both plant height (0.432) and root vitality (0.559***). This confirms that the yield benefit is predominantly indirect, mediated through a cascade of improvements in the soil-plant system.

Physiologically, MNBW irrigation markedly enhanced root activity (52.46-277.65% increase compared to CK) and free proline content, indicated improved stress resistance similar to (Yan et al., 2020). The quadratic response of root vitality to DO, peaking at 5.0 mg/L, suggests an optimal oxygen threshold, beyond which benefits diminish due to potential gas disturbance effects (Bhattarai et al., 2005; Zhou et al., 2019).

In conclusion, MNBW subsurface drip irrigation represents a scientifically grounded approach to address water scarcity and soil hypoxia challenges in alfalfa production. By simultaneously improving soil biology, root function, and plant physiology, MNBO enhances yield and resource efficiency. The identified optimal parameters provide immediate guidance for farmers, though site-specific adjustments remain essential for broader scalability across different regional conditions.

## 5 Conclusions

This study demonstrates that Micro-Nano Bubble Oxygenation in subsurface drip irrigation enhances alfalfa yield by improving soil aeration. The treatment promoted soil enzyme activities and microbial abundance, which in turn strengthened root vitality and plant stress resistance. Structural equation modeling revealed that yield improvement was primarily driven by an indirect pathway: irrigation and oxygen availability directly enhanced soil biological properties, which subsequently improved root function and plant growth, ultimately increasing yield. he optimized parameters—5.0 mg/L dissolved oxygen with 25 mm irrigation per event—effectively elevated soil oxygen levels without inducing gas disturbance, thereby sustaining microbial and root activity while avoiding over-aeration. These findings provide a scientifically sound strategy to mitigate root zone hypoxia in perennial forage crops under water-limited conditions, integrating water conservation with productivity enhancement.

## Data Availability

The raw data supporting the conclusions of this article will be made available by the authors, without undue reservation.

## Supplementary material

Supplementary Table 1 Annual irrigation specifications applied to the experimental treatments. *: Irrigations were triggered when the soil water content of W2O1 decreased to 60% of the field capacity. Therefore, the annual total amount and frequency are averages based on actual field conditions. The control treatment (CK) received conventional flood irrigation four times per year at 80 mm per application, as per local practices.
